# DHA Affects Microtubule Dynamics Through Reduction of Phospho-TCTP Levels and Enhances the Antiproliferative Effect of T-DM1 in Trastuzumab-Resistant HER2-Positive Breast Cancer Cell Lines

**DOI:** 10.3390/cells9051260

**Published:** 2020-05-19

**Authors:** Silvia D’Amico, Ewa Krystyna Krasnowska, Isabella Manni, Gabriele Toietta, Silvia Baldari, Giulia Piaggio, Marco Ranalli, Alessandra Gambacurta, Claudio Vernieri, Flavio Di Giacinto, Francesca Bernassola, Filippo de Braud, Maria Lucibello

**Affiliations:** 1 National Research Council of Italy, Institute of Translational Pharmacology (IFT-CNR), 00133 Rome, Italy; silvia.damico@ift.cnr.it (S.D.); ewa.krasnowska@ift.cnr.it (E.K.K.); 2 UOSD SAFU, Department of Research, Diagnosis and Innovative Technologies, IRCCS-Regina Elena National Cancer Institute, 00144 Rome, Italy; isabella.manni@ifo.gov.it (I.M.); giulia.piaggio@ifo.gov.it (G.P.); 3 Tumor Immunology and Immunotherapy Unit, IRCCS-Regina Elena National Cancer Institute, 00144 Rome, Italy; gabriele.toietta@ifo.gov.it (G.T.); silvia.baldari@yahoo.it (S.B.); 4 Department of Experimental Medicine, University of Rome "Tor Vergata", 00133 Rome, Italy; ranallim@uniroma2.it (M.R.); gambacur@uniroma2.it (A.G.); bernasso@uniroma2.it (F.B.); 5 Medical Oncology Department, Fondazione IRCCS Istituto Nazionale dei Tumori, 20133 Milan, Italy; Claudio.Vernieri@istitutotumori.mi.it (C.V.); Filippo.DeBraud@istitutotumori.mi.it (F.d.B.); 6 IFOM, the FIRC Institute of Molecular Oncology, 20139 Milan, Italy; 7 Department of Neuroscience, Università Cattolica del Sacro Cuore, 00168 Roma, Italy; digiacintoflavio@gmail.com; 8 Oncology and Hemato-Oncology Department, University of Milan, 20122 Milan, Italy

**Keywords:** phospho-TCTP, DHA, T-DM1, HER2-positive breast cancer

## Abstract

Trastuzumab emtansine (T-DM1) is an anti-human epidermal growth factor receptor 2 (HER2) antibody-drug conjugated to the microtubule-targeting agent emtansine (DM1). T-DM1 is an effective agent in the treatment of patients with HER2-positive breast cancer whose disease has progressed on the first-line trastuzumab containing chemotherapy. However, both primary and acquired tumour resistance limit its efficacy. Increased levels of the phosphorylated form of Translationally Controlled Tumour Protein (phospho-TCTP) have been shown to be associated with a poor clinical response to trastuzumab therapy in HER2-positive breast cancer. Here we show that phospho-TCTP is essential for correct mitosis in human mammary epithelial cells. Reduction of phospho-TCTP levels by dihydroartemisinin (DHA) causes mitotic aberration and increases microtubule density in the trastuzumab-resistant breast cancer cells HCC1954 and HCC1569. Combinatorial studies show that T-DM1 when combined with DHA is more effective in killing breast cells compared to the effect induced by any single agent. In an orthotopic breast cancer xenograft model (HCC1954), the growth of the tumour cells resumes after having achieved a complete response to T-DM1 treatment. Conversely, DHA and T-DM1 treatment induces a severe and irreversible cytotoxic effect, even after treatment interruption, thus, improving the long-term efficacy of T-DM1. These results suggest that DHA increases the effect of T-DM1 as poison for microtubules and supports the clinical development of the combination of DHA and T-DM1 for the treatment of aggressive HER2-overexpressing breast cancer.

## 1. Introduction

HER2-positive breast cancer (HER2+ BC), which represents about 25–30% of breast cancers, is characterized by the overexpression of the human epidermal growth factor receptor 2 (HER2/neu), a tyrosine kinase receptor (RTK). Therapies with monoclonal antibodies of high affinity specific for the HER2 receptor, or combinations of multiple anti-HER2 antibodies, have led to a significant improvement in therapeutic response; despite this, many patients develop resistance to therapy [1,2,3]. 

Trastuzumab emtansine (T-DM1) is an anti-HER2 antibody-drug linked to the anti-mitotic agent emtansine (DM1). T-DM1 treatment provides significant clinical benefit in breast cancer patients previously treated with chemotherapy and HER2-directed therapy, and in patients with HER2-positive early breast cancer who had residual invasive disease after completion of neoadjuvant therapy [4,5,6,7]. However, some patients may develop disease progression [8,9]. Identification of novel combination therapies for T-DM1 represents a major challenge to improve treatment effectiveness and to delay or prevent acquired resistance to HER2 inhibition.

Recently, we found that high levels of the nuclear phosphorylated form of Translationally Controlled Tumour Protein (phospho-TCTP) in HER2+ BC is associated with adverse prognostic factors and with a poor clinical response to trastuzumab therapy, suggesting a possible application of phospho-TCTP as a new marker for breast cancer [10].

TCTP is a highly conserved protein and it has been implicated in different physiological processes, including cell proliferation, cell shape, and resistance to stress [11,12,13,14,15]. Gene knockout studies have revealed that TCTP-deficient mice and TCTP-deficient mutants of Drosophila die early during embryogenesis, suggesting its implication in cell proliferation [12,16]. TCTP is a critical target in cancer therapy [15,17,18,19]. Moreover, TCTP has been identified as a critical regulator of the tumour suppressor p53 [14,20]. TCTP has been also described as a positive regulator of epithelial-to-mesenchymal transition [21,22]. Numerous clinical data show that TCTP overexpression is associated with tumour progression and poor clinical outcome in many poorly differentiated tumours [20,23,24,25,26]. Interestingly, in human breast cancer, high-TCTP status associates with poorly differentiated aggressive G3-grade tumours, predicting poor prognosis [20]. Moreover, TCTP may mediate many biological functions through the interaction with proteins involved in relevant function in cancer biology. Among these are: i) polo-like kinase 1 (PLK1), a member of the polo-like family of serine/threonine kinases that plays a crucial role in cell-cycle regulation during mitosis [27]; ii) Y-box-binding protein 1 (YBX1), a transcription and translation regulator protein that increase cancer cell invasiveness and spreading [28]; iii) the myeloid cell leukemia-1 protein (Mcl-1), an anti-apoptotic Bcl-2 family member [29]. Despite numerous reports suggesting important functions of TCTP in the context of tumour biology, its precise role is less clear.

Our previously reported data show that TCTP is a direct substrate of PLK1 in mammary carcinoma cells [10]. These data are in line with earlier published evidence [30,31,32]. Notably, the residues of Ser46 and Ser64 of TCTP, that are specifically phosphorylated by PLK1 [30], are located in a highly conserved intrinsically disordered loop of the protein, which contains a highly conserved TCTP signature. The phosphorylation event of TCTP by PLK1 may be critical for the activity of the protein, as it has been demonstrated that phospho-TCTP detaches from the spindle at the metaphase-to-anaphase transition [33]. 

It has been reported that TCTP regulates spindle microtubule dynamics during mitosis and its overexpression may lead to microtubule stabilization and alterations in cell morphology [34]. Conversely, TCTP phosphorylation by PLK1 increases microtubule dynamics by decreasing the microtubule-stabilizing activity of TCTP [30,33]. Consistent with this data, it has been reported that TCTP phosphorylating activity is low throughout the cell cycle, but increases in mitosis [11].

All together, these data suggest that TCTP may be a crucial player in mitotic processes and a fine equilibrium between the phosphorylated and non-phosphorylated forms of TCTP is required for maintaining the dynamic state of microtubules.

TCTP is a target of dihydroartemisinin (DHA) [10,35,36,37]. DHA, the active metabolite of Artemisinin, was discovered as an anti-malarial agent by Dr. Youyou Tu, who was awarded the 2015 Nobel Prize in Physiology or Medicine [38]. Notably, it has been reported that microarray-based mRNA expression of human TCTP is correlated with sensitivity to artesunate (a derivative of artemisinin) in tumour cells, suggesting that human TCTP contributes to the response of tumour cells to the drug [36]. Today, phase 1 clinical trials are underway showing that DHA has good safety and tolerability profile after long-term administration in patients with breast cancer or carcinoma of the uterine cervix [39,40,41]. These data are compatible with those obtained in malarial patients who, on the contrary, followed pharmacological treatments for shorter times [42,43]. All together, these findings shed light on the potential use of DHA as an anti-tumour agent and support the growing interest in this drug compound [44].

We have previously shown in HER2 overexpressing breast cancer cell lines that DHA, by reducing the expression levels of the phosphorylated form of TCTP, enhances the response to treatment with drugs as doxorubicin, cisplatin and trastuzumab.

Here we have investigated, in depth, the response to DHA in combination with T-DM1 in breast cancer cells resistant to trastuzumab therapy. Our data show that the combination treatments caused growth inhibition through the induction of severe mitotic perturbations, which in turn led tumour cells into an unstable state no longer compatible with viability. 

## 2. Materials and Methods

### 2.1. Chemicals

Dihydroartemisinin was from Selleckem (Munich, D) T-DM1 was provided by Genentech (South San Francisco, CA, USA). N-Acetyl-L- Cysteine (NAC) by Sigma-Aldrich, St. Louis, MO, USA.

### 2.2. Cell Culture and Treatments

All cell lines were from ATCC. HCC1569 (ER−, Pr−, Her2+), HCC1954 (ER−, Pr−, Her2+) and BT-474 cells (ER+, Pr+/−, /Her2+) were maintained in RPMI-1640 or Dulbecco’s modified Eagle’s medium (DMEM) supplemented with L-glutamine, antibiotics and 10% heat-inactivated foetal bovine serum (FBS) all from Corning (New York, NY, USA), according to ATCC indications. MCF10A cells were maintained in Dulbecco’s modified Eagle’s medium DMEM/F-12 medium containing 5% horse serum (Thermo Fisher Scientific, Waltham, MA, USA) hydrocortisone (0.5 μg/mL) (Sigma-Aldrich), insulin (10 μg/mL) (Sigma-Aldrich), Epidermal growth factor, EGF (20 ng/mL) (Sigma-Aldrich), cholera toxin (100 ng/mL) (Sigma-Aldrich), penicillin (100 units/mL) and streptomycin (100 µlg/mL) (Corning).

Cells (at 5000 cells/cm^2^ or otherwise indicated) were cultured in a humidified incubator in an atmosphere of 5% CO_2_ at 37 °C. Before any experiment, cells were detached by mild trypsinization, washed, plated in medium containing 10% FBS, and allowed to recover for 24 h. Cells were treated with DHA dissolved in DMSO (Sigma-Aldrich) or with T-DM1. Control media contained the same amount of DMSO-vehicle (<0.1%). All cell lines were tested for mycoplasma contamination regularly using MycoAlert Mycoplasma Detection Kit (Lonza, Basel, Switzerland) and were authenticated by STR sequencing (BMR Genomics, Padoa, Italy).

### 2.3. Antibodies

Primary antibodies were purchased from commercial sources.

Immunofluorescence staining: anti-TCTP (Abcam, Cambridge, UK; #ab133568); anti-Phospho-TCTP (Ser46) (Cell Signalling Technology, Leiden, NL #5251); anti-Ki-67 (Neo Markers, Fremont, CA, USA #RM-9106-S0); anti-FLAG monoclonal antibody (DYKDDDDK tag), clone M2 (anti-FLAG M2) (Sigma-Aldrich, #F1804); anti- αTubulin (Sigma-Aldrich#T9026).

Western Blot analysis: anti-histamine releasing factor (HRF)/TCTP (MBL International, Woburn, MA, USA, #JMO99-3); anti-Phospho-TCTP (Ser46) (Cell Signalling Technology, #5251); anti- Poly(ADP-ribose)polymerase (PARP) (Cell Signalling Technology #9542); anti-cleaved caspase 3 (Cell Signalling Technology #9661); anti-phospho-protein kinase B (PKB/AKT hereafter referred as AKT, Ser473) (Cell Signalling Technology #9271); anti-AKT (Cell Signalling Technology, #9272); anti phospho-AMP-activated protein kinase (AMPK) (Thr172) (Cell Signalling Technology #2531); anti-AMPK (Cell Signalling Technology #2532); anti-cyclin B1 (Santa Cruz Biotechnology, Dallas, TX, USA, sc-245); anti-FLAG M2 (Sigma-Aldrich, #F1804); anti-phospho-Histone H2AX (Sigma-Aldrich, 05-636-25UG); anti-phospho Histone H3 (Ser10) (Merck-Millipore, #06-570); anti-p44/42 mitogen-activated protein kinase (MAPK) (Erk1/2) (Cell Signalling Technology#4695); anti-phospho p44/42 MAPK (Erk1/2) (Thr202/Tyr204)(Cell Signalling Technology, #9106); anti-β-actin (Sigma-Aldrich, A1978); anti-gliceraldeide 3-fosfato deidrogenasi (GAPDH) (Santa Cruz Biotechnology, #sc25778).

Flow cytometry analysis: purified mouse anti-human c-erbB-2 (BD Biosciences, Franklin Lakes, NJ, USA, #554300). 

### 2.4. Cell Viability Assay

The ATP content was determined luminometrically by the CellTiter-Glo Luminescent Cell Viability assay, following the instructions of the manufacturers (Promega, Madison, WI, USA). Luminescence was measured with an automatic microtiter plate reader, Victor 3 V, Wallac 1420, Multilabel Counter (Perkin Elmer, Waltham, MA, USA).

### 2.5. ROS Production Assay

Reactive oxygen species (ROS) production was determined luminometrically by the ROS-Glo H_2_O_2_ Assay, following the instructions of the manufacturers (Promega). Luminescence was measured with an automatic microtiter plate reader, Victor 3 V, Wallac 1420, Multilabel Counter (Perkin Elmer).

### 2.6. Western Blot Analysis

Cells were washed with ice-cold phosphate-buffered saline (PBS), lysed in buffer contained 50 mM TRIS-HCl, pH 7.5, 400 mM NaCl, 10% glycerol, 0.5% NP40, 1% Tryton X-100 1 mM EDTA, 1 mM EGTA 2 mM DTT. All reagents were from Sigma-Aldrich. Buffer were supplemented with a protease inhibitor cocktail (P1860-Sigma-Aldrich) and phosphatase inhibitors cocktails (Sigma-Aldrich P5726 and Sigma-Aldrich P0044). Aliquots (20–60 µg) from total cell lysate proteins were resolved on 8–15% SDS–PAGE gels and analysed by immunoblotting with the indicated antibodies followed by decoration with peroxidase-labelled anti-rabbit (Thermo Fisher Scientific) or anti-mouse immunoglobulin G (IgG) (Dako-Agilent, Santa Clara, CA, USA) respectively. Blots were developed with enhanced chemiluminescence (ECL) Westar Supernova (Cyanagen, Bologna, Italy), following the instructions of the manufacturers.

### 2.7. Colony Formation Assays

Cells were seeded in 6-well plates at low density (1000 cells/plate), and cultured both in the absence or presence of drugs as indicated, for 6 days, and then incubated for 14 to 21 days to enable colony formation, after which they were fixed with 4% formaldehyde in PBS and stained with 0.1% crystal violet (Sigma-Aldrich). Relative quantification of colony number was performed using ImageJ Software (U.S. National Institute of Health, Bethesda, MD, USA).

### 2.8. Flow Cytometry

For cell cycle studies, cells were trypsinized and fixed for 45 min in methanol/acetone 4:1. After centrifugation at 950 RPM for 10 min, cells were stained with a solution containing 100 µg/mL RNase A and 50 µg/mL propidium iodide (Sigma-Aldrich) overnight in the dark at 4 °C. For detection of HER2 surface expression, cells were trypsinized and fixed for 10 min in 1% paraformaldehyde (Sigma-Aldrich). After blocking with 2% bovine serum albumin (BSA) (Sigma-Aldrich) in PBS, cells were probed with purified mouse anti-human c-erbB-2 (BD Biosciences) overnight in the dark at 4 °C. Primary antibody detection was obtained by reaction with secondary antibody Alexa Fluor 488 conjugated with IgG (Thermo Fisher Scientific, # A28175). Flow cytometry analysis was carried out using Fluorescence-activated cell sorting (FACS) Calibur flow cytometer (BD Biosciences). The percentage of cells in each stage of the cell cycle was determined using ModFit software (BD Biosciences). The percentage of cells in Sub-G1 phase was determined using FlowJo X (Tree Star Inc., Ashland, OR, USA).

### 2.9. Immunofluorescence Staining

Cells were grown on coverslips, fixed in 4% paraformaldehyde (Sigma-Aldrich) solution for 10 min. Then, samples were permeabilized in 0.2% Triton X-100/PBS for 5 min. After blocking with 3% BSA (Sigma-Aldrich) in PBS, cells were probed with the indicated primary antibodies and with Alexa Fluor 488-conjugated anti-rabbit IgG (Thermo Fisher Scientific, #A11034) or Alexa Fluor 555-conjugated anti-rabbit IgG (Thermo Fisher Scientific, #A-21428). Nuclei were counterstained with Hoechst (Fluka Biochemika, Buchs, Switzerland). Fluorescently labelled samples were imaged using a confocal LEICA TCS SP5 microscope (Leica, Heidelberg, Germany) equipped with an argon/krypton laser. Confocal sections were acquired at 0.4 μm intervals.

Excitation/emission wavelengths were 346/460 nm for Hoechst, 488/520 nm for Alexa Fluor 488 and 555/580 nm for Alexa Fluor 555. Images are shown as three-dimensional (3D) maximal projections reconstructed from z-stacks unless otherwise indicated. Magnification: 63× zoom 5 (bar = 5 µm), 63× (bar = 25 µm) and 20× (bar = 100 µm).

### 2.10. Quantification of TCTP Distribution

To study the distribution of the protein within cells, we acquired images at high magnification 63× zoom 5 (bar = 5 µm) of tumour cell lines stained with the indicated primary antibody and Hoechst (blue) at two different spectral range. Excitation/emission wavelengths used were 346/460 nm (first channel) for chromosome detection and 555/580 nm for protein detection (second channel). At least 12 cells were analysed for each sample. Thus, we performed a semi-automatic segmentation of chromosomes and cells by applying ImageJ plugin (Trainable Weka segmentation plugin) to both channels and then excluding all the cells at the image border. From these two binary masks (one for the whole cell and another for the chromosomes), we calculated pixel by pixel the distance from chromosomes of each point of the cells, using Matlab (Mathworks, USA). Collecting all the values of the second channel intensity for each distance from the chromosome, we obtained a line profile, which shows the distribution of the protein as a function of the distance from the chromosome. Signal intensity in the second channel is directly related to the quantity of protein. These results are based on a comparison between the fluorescence intensity for treated and untreated cells. The experimental setup has been kept constant for all acquisitions for cells of the same cellular line. Fluorescently labelled samples have been imaged using a confocal LEICA TCS SP5 microscope (Leica, Heidelberg, Germany) equipped with an argon/krypton laser.

### 2.11. Quantification of Ki-67 Positive Cells

We quantified Ki-67 positive cells from the analysis of fluorescence images deriving from a sample stained with Hoechst (blue) for Nuclei (first channel) and Ki-67 (red) (second channel). Excitation/emission wavelengths used were 346/460 nm for first channel and 555/580 nm for second channel. The signals from these fluorophores, acquired in two different spectral channels, allowed to contextually count the whole population of cells and distinguish the KI-67 positive cells. The count and segmentation of the whole cell population from the signal in the first channel have been performed using a custom made program of image analysis (Matlab, Mathworks, USA) based on a Gaussian filter for the background remotion and a watershed algorithm for cell segmentation. The algorithm produced a binary mask of the cells in the original image. By applying this mask to the image of the second channel, we calculated a mean value of signal intensity for each cell, and then distinguished two different groups within the whole population, through the set of a threshold value in intensity. In each group, we analysed: i) 2380 cells for MCF10A-pBabe; ii) 3612 cells for MCF10A-AATCTP; iii) 2483 cells for MCF10A-WTTCTP (right panel).

### 2.12. Quantification of Microtubule Density

For the quantitative analysis of the microtubule density, we analysed the fluorescence images obtained by staining the sample with Hoechst (blue) and αTubulin (green). Excitation/emission wavelengths used were 346/460 nm (blue) and 488/520 nm (green). At least 500 cells were analysed for each sample. For quantification analysis, we firstly removed the background to highlight the strong signal from tubulin by applying a Gaussian filter to images, and then we subtracted this image from the original one. Afterwards, we applied a threshold to segment the tubulin and create a binary image of these structures. Finally, we compared this mask to the mask of the whole area in the image field, which is covered by cell. Thus, to obtain a quantification of tubulin density, we divided these two values, obtaining the fraction of pixels, which appears to be crossed by tubulin structures. 

### 2.13. Growth Curve

Cells (5 × 10^4^) were seeded in each well of 6-well plate. The cell number for each cell line was counted each day for a 6-day time course; this number has been normalized on cell number counted 24 h after seeding to allow cells to recover from the trypsinization. Cell numbers have been counted in triplicate in three independent experiments. Doubling times were determined by fitting the data points to the exponential growth function in GraphPad Prism 5.01 software.

### 2.14. Vector Construction

Plasmids pcDNA3-TCTP were constructed as previously described [10].

### 2.15. Mutagenesis

Mutagenesis of sites of phosphorylation of TCTP Ser46 and Ser64 were performed as previously described [10].

### 2.16. Recombinant Retroviral Vectors

The PCR amplified fragments wild type (WT) and Ser46Ala Ser64Ala double mutant (AA) were cloned in *EcoR1* site of pBABE-Puro retroviral vector to obtain FLAG-TCTP-pBABE and FLAG-AA-TCTP-pBABE. All constructs were confirmed by DNA sequence analysis.

### 2.17. Cell Transfection

Retroviruses were produced by transfection of Phoenix-Ampho packaging cells with pBABE-puro, AA-TCTP-pBABE, and WT-TCTP-pBABE using Lipofectamine 2000 (Invitrogen, Carlsbad, CA, USA). At 48 h after transfection, supernatants containing the retroviral particles were collected and frozen at −80 °C until use. MCF10A cells were infected with diluted supernatant in the presence of 8 µg/mL Polybrene (Sigma-Aldrich) overnight, and cells containing the pBABE, AA-TCTP-pBABE, and WT-TCTPpBABE constructs were selected with puromycin (1 µg/mL) (Sigma-Aldrich) 48 h after infection. After 10 days in selective medium, the three pools referred to empty vector (MCF10A-pBABE), the wild type TCTP protein (WT-TCTP), the Ser46Ala Ser64Ala double mutant TCTP (AA-TCTP), were isolated. The puromycin selective pressure was removed 24 h before experimental procedures.

### 2.18. Evaluation of Cell Sensitivity to Combined Treatment

Cells were plated in triplicate in 96-well and treated with DHA, T-DM1, and with the DHA/T-DM1 combination. Growth inhibition was calculated as the percentage of viable cells compared to untreated cells by the CellTiter-Glo Luminescent Cell Viability assay (Promega, Madison, WI, USA) The CompuSyn software program has been used to calculated synergistic, additive or antagonistic effects. This program is based on the Median-Effect Principle (Chou) and the Combination Index–Isobologram Theorem (Chou-Talalay) [45]. Because all terms in the equations are ratios, all the dose units become dimensionless quantities. Drug can be different units. The combination index (CI) indicates a quantitative measure of the degree of drug interaction in terms of synergistic (CI < 1), additive (CI = 1) or antagonistic effect (CI > 1). DRI is the dose-reduction index and it is a measure of how many-fold the dose of each drug in a synergistic combination may be reduced at a given effect level compared with the doses of each drug alone.

### 2.19. Immunodeficient Mice Study

We generated HCC1954 cells expressing luciferase in order to implement bioluminescent imaging analysis to follow breast tumour growth in small animal models in vivo. Briefly, HCC1954 cells were transduced at multiplicity of infection MOI 10 with a third-generation self-inactivating lentiviral vector expressing firefly luciferase [46]. Six-week-old CB17SCID female mice were purchased from Charles River (Calco, Italy) and housed with laboratory chow and water available ad libitum. 

A cell-line derived orthotopic xenograft model of breast cancer was established by mammary gland implantation of 5 × 10^5^ HCC1954 luciferase-expressing cells. Mice were regularly palpated and tumour dimensions were measured once a week using a digital calliper. Moreover, tumour cell engraftment and early detection of tumour growth was assessed by longitudinal bioluminescent analysis (BLI). BLI analysis has been performed using the IVIS^®^ Lumina II equipped with the Living Image^®^ software for data quantification (PerkinElmer). Animals were sedated and D-luciferin (PerkinElmer) dissolved in PBS (150 mg/kg body weight) was administered i.p. 10 min before analysis [47]. Photons emitted from luciferase expressing HCC1954 cells implanted into the animals were collected with final accumulation times ranging from of 1 s to 1 min, depending on the intensity of the bioluminescence emission. All animal experiments were conducted in accordance with institutional guidelines, in the full observation of the Directive 2010/63/UE.

### 2.20. Statistical Analysis

All experiments were done at least three times unless otherwise indicated. The results are presented as means ± SD. Results were analysed using a Mann–Whitney test. One-way ANOVA followed by the Bonferroni test using the PRISM GraphPad software was used in the analysis of three or more data sets. Differences were considered significant for *P* < 0.05 and highly significant for *P* < 0.01 and *P* < 0.001

## 3. Results 

### 3.1. DHA Affects Mitosis of HER2+ BC Cell Lines with Aberrant PI3K/AKT Signalling

We investigated the effect of DHA on HER2+ breast cancer cells resistant to trastuzumab. Since PI3KCA mutations and/or loss of phosphatase and tensin homolog (PTEN) have been associated with a lower response to trastuzumab and chemotherapy [1,48,49], we chose the HCC1954 cell line characterized by a mutation in the catalytic domain (H1047R) of *PI3KCA* and the HCC1569 cell line, which is PTEN-null. Both cell lines showed resistance to trastuzumab or pertuzumab therapy, in line with the data in literature [50], whereas HER2-positive breast cancer BT-474 cell line was sensitive to antibody therapy (Supplementary Figure S1). 

**Figure 1:**
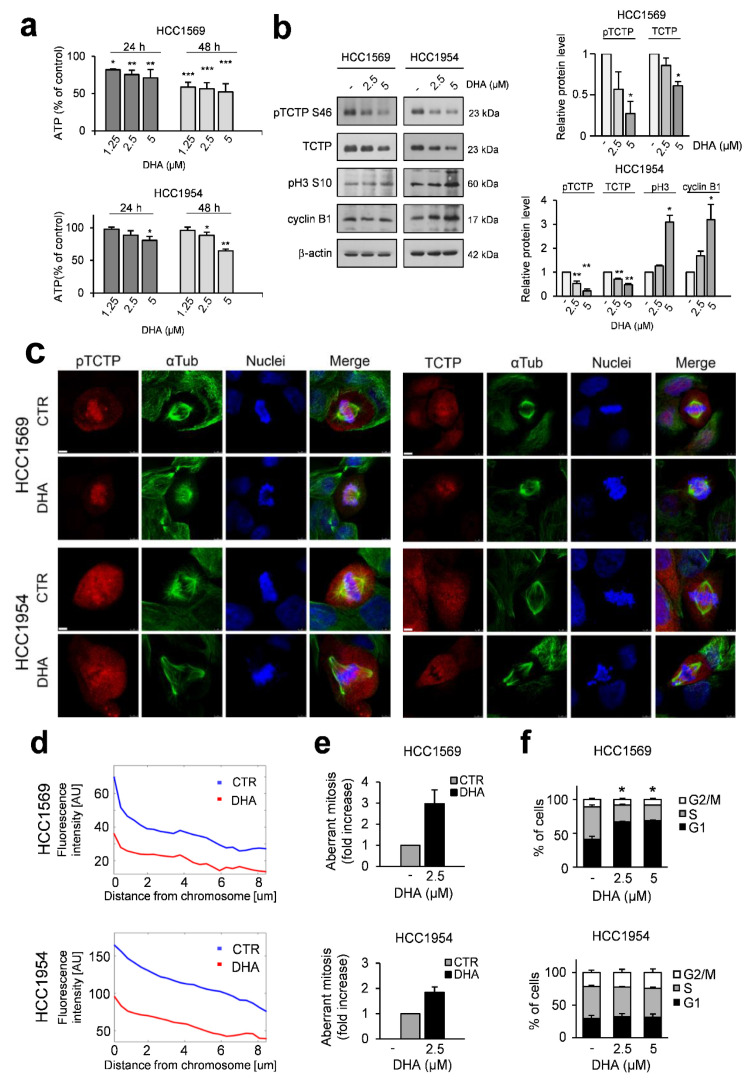
Dihydroartemisinin (DHA) induces aberrant mitosis in HER2-positive breast cancer (HER2+ BC) cell lines. (**a**) Cell viability was assessed by ATP assay. Data are expressed as the percentage of viable cells relative to controls. Values represent the mean ± SD, n = 3. (**b**) Western Blot analysis of the indicated proteins in cell lysates of cells treated with DHA 24 h. β-actin was used as loading control (right panel). For densitometric analysis, the intensity of each band was normalized to the respective β-actin (left panel). (**c**) Immunofluorescence detection of phospho-Translationally Controlled Tumour Protein (pTCTP S46) (red) or TCTP (red) and αTubulin (green) in cells treated with DHA at 2.5 µM for 24 h. Nuclei were stained with Hoechst (blue). The overlay of the three fluorochromes is shown (Merge), bar = 5 µm. Data from a representative experiment of three with similar results are shown. (**d**) Quantification of p-TCTP. Cells were treated as described in (**c**). The line profile shows the distribution of pTCTP as a function of the distance from the chromosome. The quantification was performed as described in Materials and Methods. (**e**) Bar graphs show relative quantification of fraction of cells with aberrant mitosis. At least 1000 cells were analysed in each group. Values represent the mean ± SD. (**f**) Cell cycle distribution in cells treated with DHA for 24 h. Values represent the mean ± SD, *n* = 3. * = *p* < 0.05, ** = *p* < 0.01, *** = *p* < 0.001 vs. control.

Cells were grown in the presence or absence of DHA. As shown in Figure 1 (panel a), DHA induced a mild but significant inhibition of cell viability during the first 24–48 h at concentrations ranging from 1.25 to 5 µM, which are achievable in the clinic [40,42,43,51]. Western blot analysis also showed that DHA at these concentrations induced a reduction of both total and phospho-TCTP levels, in accordance with our previous data (Figure 1b) [10]. PLK1 inhibition impairs TCTP phosphorylation [10,52] as does DHA. Since PLK1 is a master mitotic regulator [27], we investigated the impact of DHA on mitotic cells. To this end, we performed a morphological analysis in both cell lines after 24 h of exposure to DHA. Control mitotic cells were characterized by a bright peri-chromosomal localisation of both phospho-TCTP (Figure 1c, left panel), and TCTP (Figure 1c, right panel), in accordance with data from the literature [31]. The signal intensity of phospho-TCTP was higher around the chromosomes than in the rest of cells, only in untreated cells, as indicated by a quantification analysis of peri-chromosomal localization of phospho-TCTP in both DHA-treated and untreated cells (Figure 1d). No differences in the signal intensity of TCTP were observed between untreated and treated cells (data not shown).

Interestingly, DHA-treated cells showed a significant increase of aberrant spindle structures (Figure 1e), such as multipolar or monopolar spindles, or chromosome misalignments (Figure 1c). The treatment did not arrest the progression of these cells through G2 and mitosis (Figure 1f). However, the cell cycle distribution analysis showed that DHA blocked the G1/S transition of a small, yet remarkable, percentage of HCC1569 cells (Figure 1f, upper panel). In addition, Western blot analysis showed that DHA induced an increase of Cyclin B1 and phospho-Histone-H3 in HCC1954 cells, thus suggesting that a delay in mitotic progression could be induced by treatment (Figure 1b). Altogether, these data show that aberrant mitosis could be induced by an early treatment with DHA in aggressive HER2+ BC cell lines and a reduction of the peri-chromosomal localization of phospho-TCTP level occurs at the same time.

### 3.2. DHA Induces a Decrease in AKT Phosphorylation Levels and DNA Damage Through the Increase of ROS in HER2+ BC Cell Lines

**Figure 2:**
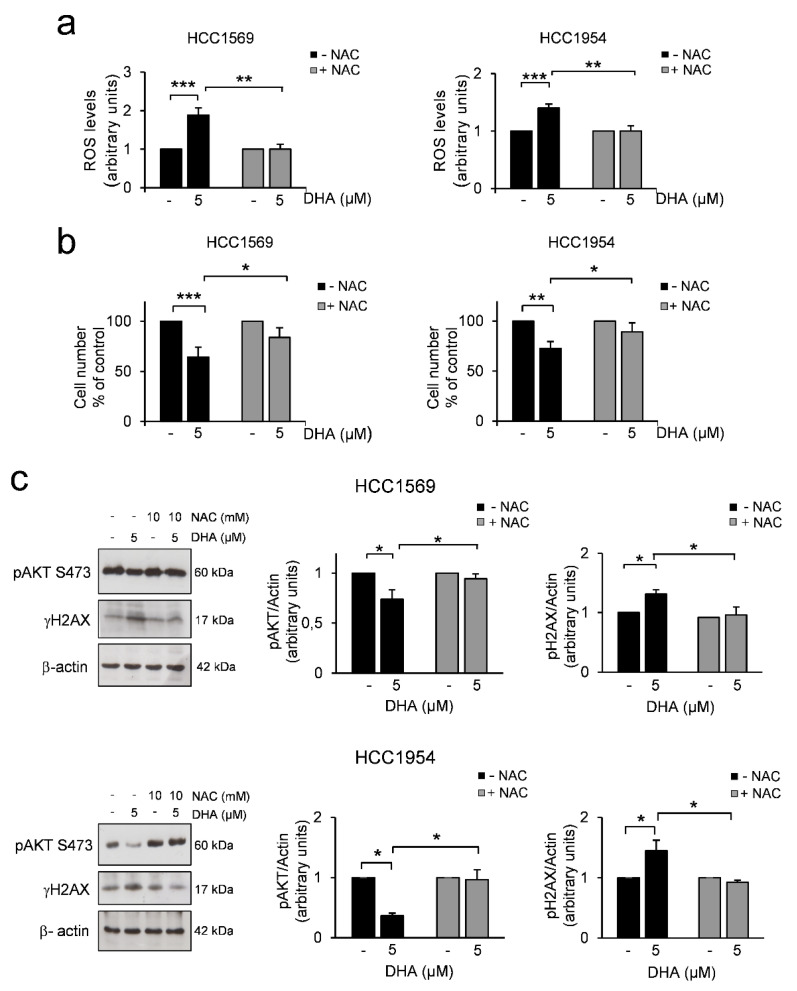
DHA induces oxidative stress in HER2+ BC cell lines. (**a**) Reactive oxygen species (ROS) production was assessed in cells pretreated with N-Acetyl-L- Cysteine (NAC) (10mM) followed by treatment with DHA for 24 h. ROS production was measured at the end of incubation time. Data are expressed as ROS levels relative to controls. n = 5. (**b**) Trypan blue exclusion assay was performed in cells treated as described in a). Data are expressed as the percentage of viable cells relative to controls. Values represent the mean ± SD, n = 5. (**c**) Western Blot analysis of the indicated proteins in HCC1569 (upper panel) and HCC1954 (lower panel) cells treated as described in a). β-actin was used as loading control. For densitometric analysis, the intensity of each band was normalized to the respective β-actin. * = *p* < 0.05, ** = *p* < 0.01, *** = *p* < 0.01.

Then we investigated whether DHA could prevent phosphorylation on AKT’s activation sites, as it has been demonstrated that active AKT is a crucial player in the downstream HER2 signalling pathways [1]. To this end, we evaluated the level of active AKT, in both cell lines after 24 h of DHA treatment. Western blot analysis of the total cell lysates showed that DHA induced a decrease in AKT phosphorylation levels (Figure 2c). DHA contains an endoperoxide moiety [53] that generated reactive oxygen species (ROS) in the first hours of treatment (Figure 2, panel a). To determine the implication of oxidative stress in the cytotoxicity of DHA, cells were pretreated with the ROS scavenger N-Acetyl-L-Cysteine (NAC) for 3 h followed by treatment with DHA. DHA-induced increase in ROS levels was abrogated by NAC (Figure 2a). In addition, NAC administration partially protected the cells from the anti-proliferative effect of DHA (Figure 2b), thus indicating that DHA cytotoxicity may be mediated at least in part by increase of ROS.

Then we investigated whether the phosphorylation of AKT can also be regulated by ROS. Notably, NAC reversed the inhibition of AKT phosphorylation induced by DHA treatment in both cell lines (Figure 2c). Since the increase of ROS levels may induce oxidative DNA damage in DHA-treated cells, we examined the phosphorylation levels of histone 2AX (γH2AX), as a marker of DNA double-strand breaks and genomic instability, in both cell lines after 24 h of exposure to DHA. A mild increase of H2AX phosphorylation at Ser139 was clearly detectable at 24 h of DHA treatment in both cell lines and it was reverted by NAC (Figure 2c). 

These data show that, beyond the induction of mitotic perturbations, through the increase of oxidative stress, DHA could further damage cancer cells and, thus, might render them more sensitive to a subsequent treatment.

### 3.3. Phosphorylation of TCTP is Required for Correct Mitotic Progression in Human Mammary Cells

**Figure 3:**
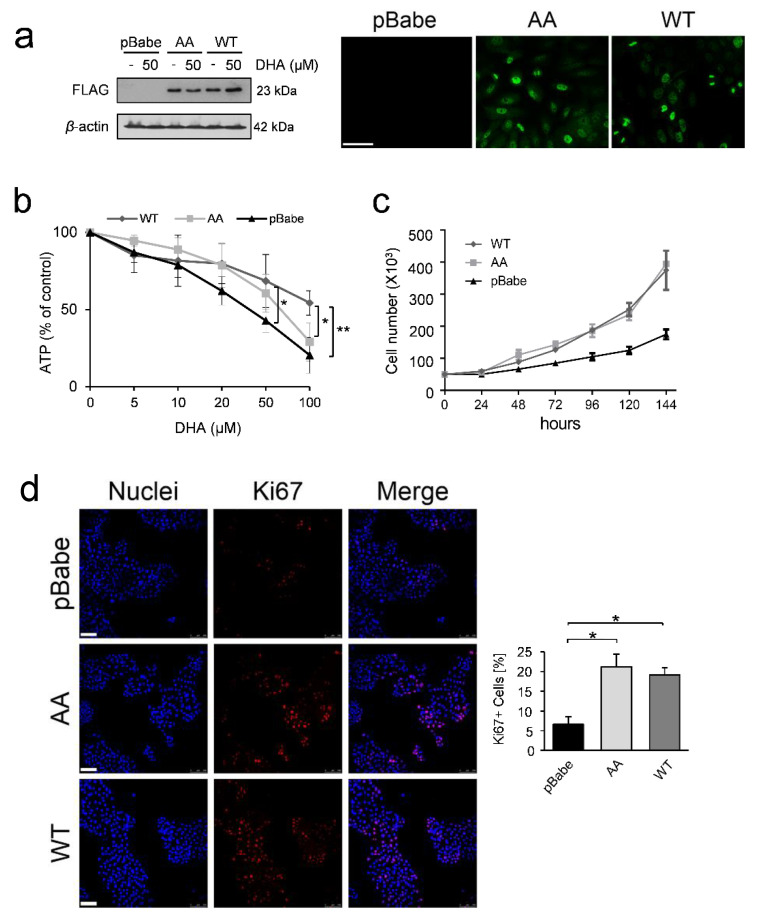

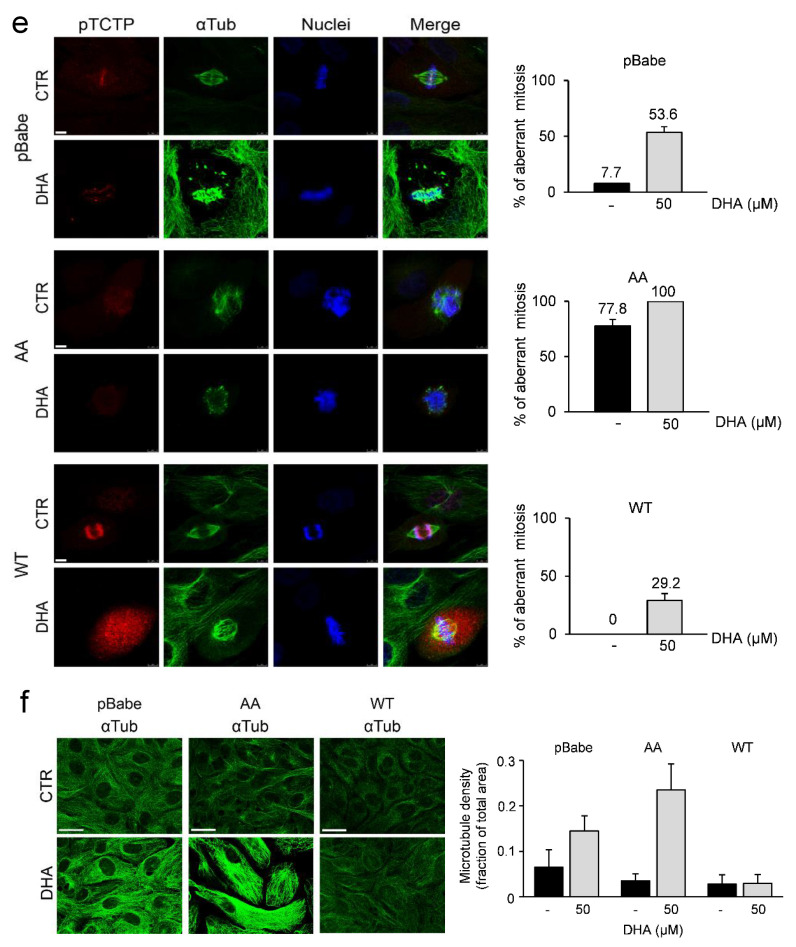
Effects of DHA on mitosis in human mammary cells. (**a**) Western blot analysis of FLAG-tagged TCTP protein in cell lysates of MCF10A-pBabe (pBabe), MCF10A-AATCTP (AA), MCF10A–WTTCTP (WT) cells after 6 days of exposure to DHA. β-actin was used as loading control (upper panel). Immunofluorescence detection of FLAG (green) fusion proteins, bar = 25 μm. (**b**) Cell viability was assessed by ATP assay in cells treated with DHA for 6 days. Data are expressed as the percentage of viable cells relative to controls. Values represent the mean ± SD, n = 3. * = *p* < 0.05, ** = *p* < 0.01. (**c**) Growth curve for all cell clones. (**d**) Immunofluorescence detection of Ki-67 (red). Nuclei were stained with Hoechst (blue). The overlay of the two fluorochromes is shown (Merge). Images are shown as single slice of a projection, bar = 100 μm. Data from a representative experiment of two with similar results are shown (left panel). Quantification of Ki-67 positive cells was performed as described in Materials and Methods (right panel). Values represent the mean ± SEM, * = *p* < 0.05. (**e**) Immunofluorescence detection of phospho-TCTP (pTCTP S46) (red) and αTubulin (green). Nuclei were stained with Hoechst (blue). The overlay of the three fluorochromes is shown (Merge). Cells were treated with DHA (50 µM) for 6 days. Bar = 5 µm). Data from a representative experiment of three with similar results are shown (left panel). Quantification of fraction of cells with aberrant mitosis. At least 1000 cells were analysed in each group. Values represent the mean ± SD. (right panel). (**f**) Immunofluorescence detection of αTubulin (green) in cells treated as described in (**e**), bar = 25 µm), left panel. Quantitative analysis of the microtubule density. The quantification was performed as described in Materials and Methods. Values represent the mean ± SD, right panel.

To investigate the role of phospho-TCTP in cells committed to mitosis, we performed a series of experiments using the MCF10A cells, a non-tumorigenic human mammary cell line, which expresses low phospho-TCTP and TCTP levels (as previously demonstrated [10]), in which we overexpressed WT FLAG-tagged TCTP protein (WT) or a non-phosphorylatable mutant (AA) TCTP protein. To this aim, we carried out a retroviral infection to establish stable MCF10A subclones expressing: 1) empty vector, hereafter called MCF10A-pBabe; 2) wild-type TCTP protein (WT-TCTP), hereafter called MCF10A-WTTCTP; 3) Ser46Ala Ser64Ala double mutant TCTP (AA-TCTP), hereafter called MCF10A-AATCTP. As shown in Figure 3a, the expression of both forms (WT-TCTP and AA-TCTP) was verified assessing the level of tagged proteins by Western Blot (Figure 3a, left panel) and immunofluorescence analysis (Figure 3a, right panel). 

We compared MCF10A-pBabe cells to MCF10A-AATCTP and MCF10A-WTTCTP cells for their resistance to DHA. To this end, all cell clones were grown in the absence or presence of different concentrations of DHA for 6 days. As shown in Figure 3b, DHA induced an inhibitory effect in all three cell clones at significantly higher concentration than those active on cancer cells (>10 µM) (Table 1), suggesting that DHA can target cancer cells without harming healthy tissues. Overexpression of TCTP protected cells against DHA induced-cytotoxicity, as indicated by the fold increase in half-maximal effective concentration EC_50_ values reported in Table 1.

We also found that overexpression of TCTP conferred a growth advantage. A growth curve analysis clearly demonstrate that doubling time of MCF10A subclones overexpressing both WTTCTP (46.79 h) and AATCTP (47.36 h) was shorter than that of MCF10A pBabe (71.88 h), indicating a more rapid proliferation (Figure 3, panel c). Moreover, quantification immunofluorescence analysis showed that both MCF10A-AATCTP and MCF10A-WTTCTP cells had higher expressions of Ki-67, a marker of cell proliferation, which is not detected in non-cycling cells (Figure 3d). 

However, the overexpression of the non-phosphorylatable form of TCTP leads to an increase of a phenotype characterized by mitotic aberration [30]. In line with this data, control MCF10A-AATCTP cells showed aberrant mitotic figures, as shown by an immunofluorescence analysis (Figure 3e, left panel) and by a quantification analysis (Figure 3e, right panel). DHA at a concentration of 50 µM induced in these cells a severe damage, as indicated by a quantitative analysis showing an increase in microtubule mass mainly in MCF10A-AATCTP cells **(**Figure 3f). In contrast, we did not find any relevant alterations to the spindle morphology or to microtubule density between treated and non-treated cells in MCF10A-WTTCTP (Figure 3e,f) suggesting that expression of the phosphorylated form of TCTP is required for correct mitosis. DHA induced an increase in ROS levels in all cell clones. However, the increase in ROS levels after treatment was significantly higher in MCF10A-AATCTP cells when compared to MCF10A-WTTCTP cells (Supplementary Figure S2, panel a). The phosphorylation of AKT was not substantially affected by DHA and only a very light inhibition was observed in AA-MCF10 cells (Supplementary Figure S2, panel b). Moreover, DHA induced a very light increase of γH2AX levels only in MCF10A-AATCTP cells in comparison to the other cell clones (Supplementary Figure S2, panel b), thus indicating a greater vulnerability of these cells to DNA damage.

Altogether, these data show that TCTP is a crucial player in mitotic processes. By reduction of the phosphorylated form of TCTP, by mutagenesis and by DHA treatment, resulted in mitotic aberrations and increased microtubule density.

### 3.4. DHA Enhances T-DM1 Efficacy in Breast Cancer Cells Resistant to Trastuzumab Therapy

We then investigated the combinatorial effects of DHA and T-DM1 in both HCC1954 and HCC1569 cell lines. In a pilot study, we used two different protocols. In the first protocol, the effect of T-DM1 was studied in cells pre-treated with DHA and then exposed to T-DM1, while in the second protocol the drugs were administered at the same time. However, no remarkable differences in term of efficacy and CI values were obtained between the two protocols in both cell lines (data not shown). Since DHA induced mitotic aberration during the first 24 h of exposure, we decided to follow the first protocol.

HCC1954 cells were sensitive to both T-DM1 and DHA treatments as indicated by parameters from the dose-response curves obtained with various concentrations of these drugs (Table 2). Notably, T-DM1 showed a sharp increase in the slope of a dose-response curve, indicating a high cytotoxicity above the EC_50_ value. 

**Figure 4:**
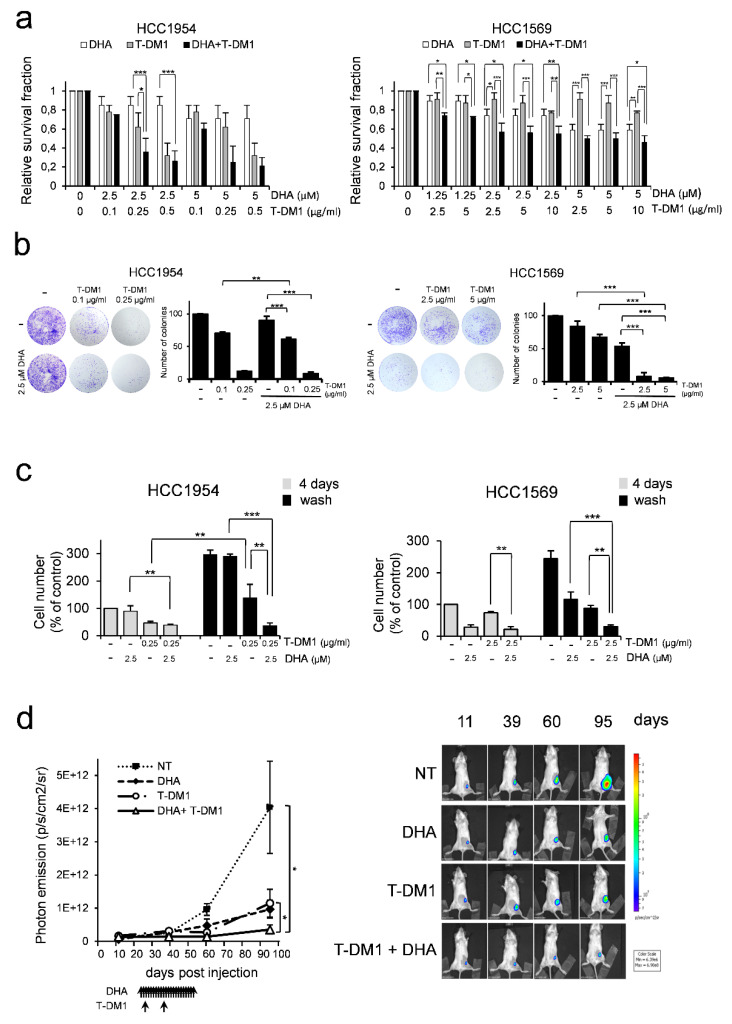
T-DM1 when combined with DHA is more effective in killing cancer cells. (**a**) Cell viability was assessed by ATP assay in cells pre-treated with DHA for 24 h and then treated with T-DM1 for 5 days. Relative survival fraction after treatment, control cells were set to “1”. Values represent the mean ± SD, *n* = 3 * = *p* < 0.05, ** = *p* < 0.01, *** = *p* < 0.001 (**b**) Colony formation assay. Cells were treated as described in (a). A representative experiment of three with similar results is shown. Bar graphs show quantification analysis. Values represent the mean ± SD, *n* = 3. ** = *p* < 0.01, *** = *p* < 0.001. (**c**) Trypan blue exclusion assay was performed in cells pre-treated with DHA for 24 h and then treated with T-DM1 for 3 days (grey columns). On day 4, cells were washed with fresh media and further incubated for additional 4 days (black column). Data are expressed as the percentage of viable cells relative to controls. Values represent the mean ± SD, *n* = 3. * = *p* < 0.05, ** = *p* < 0.01, *** = *p* < 0.001. (**d**) Quantification of tumour xenografts growth by bioluminescence imaging. Luciferase-expressing HCC1954 cells were implanted into the mammary gland of CB17SCID mice. After 22 days animals were treated with: (1) vehicle; (2) DHA, administrated intraperitoneally (i.p.) 5 days per week, for 4 weeks, in a single daily dose of 25 mg/kg; (3) T-DM1, intravenously (i.v.), once every twelve days for a total of two injections at doses of 10 mg/kg; (4) the combination of DHA and T-DM1. Bioluminescence was detected in mice 11, 39, 60 and 95 days after tumour inoculation. Mean ± SEM is shown. Photon emission is measured as photons/sec/cm2/steradian (N = 5), *P* < 0.05, Mann Whitney test (left panel). Bioluminescence of a representative mouse at the indicated days after tumour inoculation. The intensity of light emission from the animals is represented in pseudo-colour scaling (right panel). * = *p* < 0.05.

Drugs were tested at different concentrations in order to find out which ratio yielded a better response. Based on the values of EC_50_ obtained from the dose-response curve of each drug, a first study was carried out in HCC1954 cells by mixing the two drugs (DHA and T-DM1) at various ratio from 50:1 to 10:1 (data not shown). The two-drug combination at a ratio of 10:1 yielded synergistic inhibition (Table 2). We have also extend the studies at a non-constant combination ratio (e.g., keep DHA at constant dose and vary the dose of T-DM1). DHA was used at concentration ranging from 1.25 to 5 µM (Table 2). Interestingly, the two-drug combination was more efficacious in inhibiting cell growth when cells were treated with DHA at 2.5 µM and T-DM1 at 0.25 µg/mL (Table 2, and Figure 4a, left panel). 

The HCC1569 cell line was less responsive to T-DM1 treatment when compared to HCC1954 cells, as indicated by the EC_50_ value (EC_50_ =101.70 ± 44.8) reported in Table 2. We chose an arbitrary ratio of 1:1 of drug combinations in order to identify the minimum effective dose of each drug. Results obtained from drug combination studies are shown in Table 2 and Figure 4a (right panel). Overall, we found that DHA synergized with T-DM1, as indicated by the CI values less than 1 (Table 2). Interestingly, analysis of the dose reduction index (DRI) indicated that addition of DHA to T-DM1 allowed a dose-reduction for T-DM1 in both cell lines (Table 2). For instance, the addition of DHA at 2.5 µM to T-DM1 allowed up to 3-fold and ~10-fold reduction of T-DM1 in HCC1954 cells and HCC1569 cells, respectively. 

The inhibition of cell growth induced by the combination of DHA and T-DM1 has been also confirmed by colony assay experiments in both cell lines (Figure 4b).

To further test the efficacy of the combination of DHA and T-DM1, we performed washout experiments. Cells were cultured at low density (1500 cells/cm^2^) to maintain them in exponential growth. HCC1954 cells and HCC1569 cells were pre-treated with DHA at 2.5 µM and then treated with T-DM1 at 0.25 µg/mL and 2.5 µg/mL, respectively, for 3 days. This was followed by the removal of the drugs from the media and by a further incubation for additional 4 days. At the end of incubation time, the number of viable cells was determined by trypan blue dye exclusion assay. Figure 4c shows that a severe inhibition of cell growth was induced in both cell lines by the DHA and T-DM1 treatment. This effect persisted to a greater extent than those of each single agent upon removal of the drug-containing medium. 

We then studied the inhibitory effects of DHA, T-DM1 and the two-drug combination in vivo. An orthotopic xenograft model of breast cancer was established by implantation of luciferase-expressing HCC1954 cells in the mouse mammary gland. Tumour cell engraftment and early detection of tumour growth was assessed by BLI analysis. To determine the in vivo antitumour activity of the treatments, pharmacological administration was initiated 22 days after tumour cell inoculation, when tumour bioluminescent emission reached an average value of 8 × 10^9^ photons/sec/cm2/steradian (Supplementary Figure S3, panel a). Animals were divided into 4 experimental groups (N = 5 per group). Following treatment, mice were monitored for tumour recurrence. Within the period of follow-up, any single agent or the two-drug combination were well tolerated, with no signs of toxicity and weight loss (Supplementary Figure S3, panel b). As expected, T-DM1 treatment was effective in inhibiting tumour growth. However, the growth of the tumours resumed after having achieved a complete response (Figure 4d and Supplementary Figure S3, panel c), in line with data from literature [54,55]. In contrast, the combination DHA and T-DM1 was more efficient than each single agent on tumour inhibition throughout the observation period (Figure 4d, and Supplementary Figure S3, panel c), consistent with the results shown in Figure 4c.

Altogether, these data suggest that T-DM1 when combined with DHA is more effective in killing HER2+ BC cell lines.

### 3.5. The Effects of Two-Drug Combination on HER2-Mediated Cell Signalling

**Figure 5:**
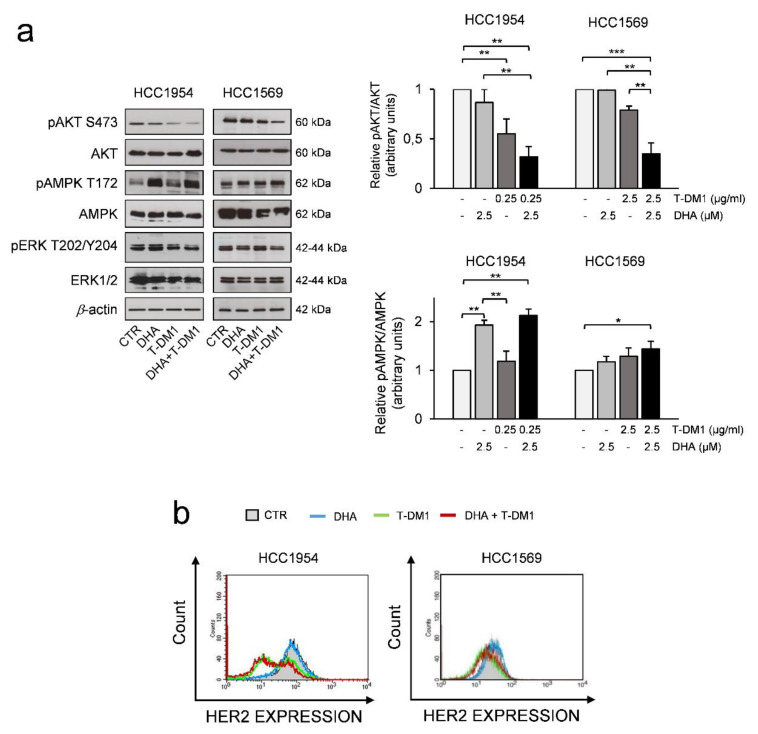
Effects of Two-drug combination on Protein kinase B (PKB/AKT hereafter referred as AKT), phospho-AMP-activated protein kinase (AMPK), and human epidermal growth factor receptor 2 (HER2) expression in HER2+ BC cell lines. (**a**) Western Blot analysis of the indicated proteins in cells pre-treated with DHA for 24 h and then treated with T-DM1 for 3 days. β-actin was used as loading control (right panel). For densitometric analysis the intensity of each band was normalized to the respective β-actin Quantification analysis was performed by using ImageJ software (left panel). (**b**) Quantitative flow cytometry (FACS) analysis of membrane HER2 expression. Cells were treated as described in (**a**) Histograms show one representative experiment of two with similar results. * = *p* < 0.05, ** = *p* < 0.01, *** = *p* < 0.01.

It has been reported that T-DM1 inhibits AKT phosphorylation in both trastuzumab-responsive and insensitive cell lines, suggesting that this effect could be mediated by the DM1 component of T-DM1 [56,57]. Therefore, we assessed the levels of activation of AKT in HCC1954 and HCC1569 cells pre-treated with DHA for 24 h and then exposed to T-DM1 for three days. Western blot analysis showed a great reduction of active AKT in cells treated with the combination of two drugs as compared to the effect induced by each single agent (Figure 5a). After four days of treatment, phospho-AKT levels were not affected by DHA (Figure 5a) and this suggests that only the T-DM1 and DHA combination was effective enough to reduce active AKT in the long-term period.

Then we investigated the effects of two-drug combination on the activation of p44/p42 MAPK (also called ERK1/2). Western blot analysis showed that the levels of phosphorylated ERK1/2 were barely reduced by the two-drug treatment. 

We also studied the phosphorylation of AMPK at its activation site Thr172. Interestingly, the two-drug treatment induced an increase of pphospho-AMPK, which was well-detectable in HCC1954 cells and, to a lesser degree, in HCC1569 cells when compared to control (Figure 5a). Interestingly, AMPK activation may play a role in mitosis and genomic stability beyond its role in the metabolic stress response [58,59] and could be activated upon T-DM1 or DHA treatment. 

One critical point in the anti-HER2 therapy is that patients can experience altered HER2 status [60,61], and loss of HER2 expression could lead to a resistant phenotype. We found that T-DM1 treatment induced a reduction of cells with the highest levels of HER2 and an enrichment of cells with low levels of HER2 in HCC1954 cells, suggesting that T-DM1 eliminated cells expressing the highest HER2 levels, but still are responsive to the treatment, in line with data from the literature [9] (Figure 5b). In contrast, the HER2 status was not affected by T-DM1 in HCC1569 cells, which were less responsive to T-DM1 treatment (Figure 5b). Altogether, these data suggest that the inhibition of phospho-AKT could be mediated by the DM1 component of T-DM1 in HER2+ BC cell lines resistant to trastuzumab. A great reduction of active AKT is observed in these cells when treated with the combination of two drugs as compared to the effect induced by each single agent.

### 3.6. DHA in Combination with T-DM1 Led to Mitotic Catastrophe

**Figure 6:**
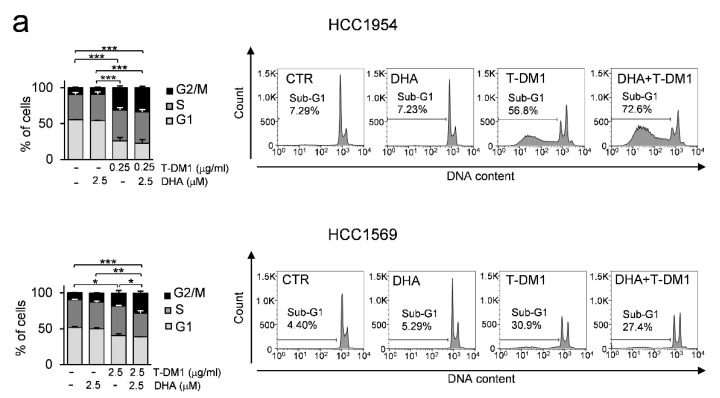

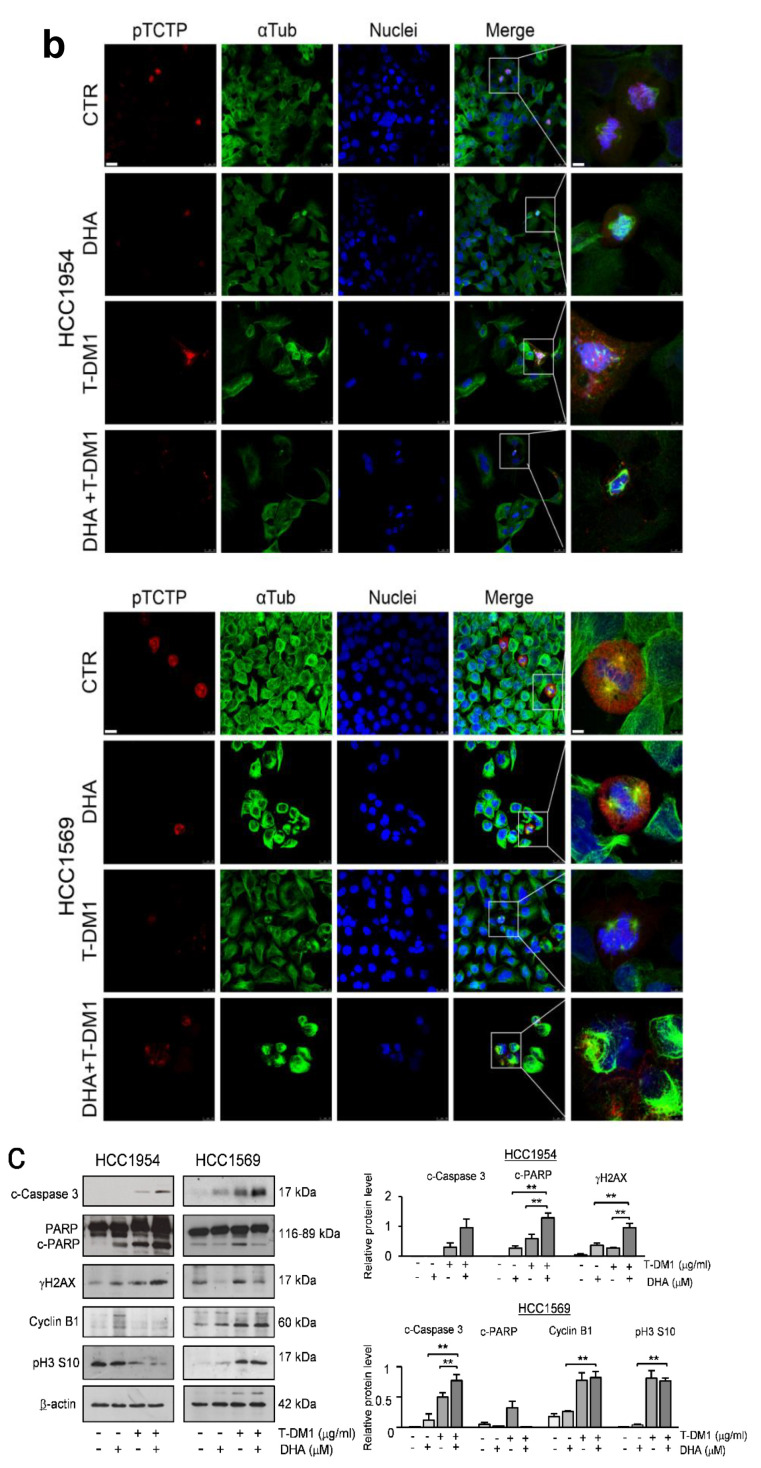
The combination of DHA and T-DM1 induces mitotic catastrophe in HER2+ BC cell lines (**a**) Cells were pre-treated with DHA for 24 h and then treated with T-DM1 for 3 days. The bar graphs show the distribution of cycling cells. The histograms show the percentage of Sub-G1 cells of one representative experiment. Values represent the mean ± SD, *n* = 3. * *p* < 0.05, ** *p* < 0.01, *** *p* < 0.001. (**b**) Cells were treated as described in (**a**). The overlay of the three fluorochromes is shown (Merge): phospho-TCTP (red) and αTubulin (green) and nuclei stained with Hoechst (blue), bar = 25 µm (left side). A greater detail of the boxed area is shown, bar = 5 µm (right side). Data from representative experiments are shown. *n* = 2. (**c**) Western Blot analysis of the indicated proteins in cell lysates of cells treated as described in a). β-actin was used as loading control (left panel). For densitometric analysis, the intensity of each band was normalized to the respective β-actin (right panel). * *p* < 0.05, ** *p* < 0.01.

Since T-DM1 is a microtubule-disrupting agent that may cause cell cycle arrest in the G2 and M phases, we evaluated its effects on cell cycle distribution when combined with DHA. The treatment of both cell lines with T-DM1 and with the DHA and T-DM1 combination inhibited cell-cycle progression, as indicated by the increased proportion of cells in G2/M phase (Figure 6a). Moreover, the treatment with T-DM1 induced a remarkable increase of cell population in sub-G1 area mainly in HCC1954 cells. The increase was even greater in HCC1954 cells exposed to the two-drug treatment, thus indicating apoptotic cells and/or fragmentation of DNA (Figure 6a). 

An immunofluorescence analysis revealed aberrant mitotic spindles in both cell lines under all treatments. Both T-DM1 and the DHA and T-DM1 combination induced the formation of disorganized microtubule structures, unstructured tubulin foci, and multiple nuclei. In addition, a significant (approximately 2-fold) increase in density of microtubules was clearly found when HCC1569 cells were exposed to both DHA and DHA and T-DM1 combination, while we did not find any significant increase in density of microtubules in HCC1954 cells under all treatments (Supplementary Figure S4). Moreover, we also detected an aberrant distribution of phospho-TCTP in enlarged and morphologically altered HCC1569 and HCC1954 cells when treated with DHA and T-DM1 (Figure 6b). 

Dividing cells with a defective mitotic apparatus undergo mitotic cell death/catastrophe [62]. Western blot analysis of cyclin B1 and phosphorylated histone H3 at Ser10 (p-Histone H3), two markers of M phase, showed that they were upregulated in HCC1569 cells, thus indicating that cells were arrested in mitosis. In addition, we found that activation of caspase 3 was induced by T-DM1 treatment. Notably, the increase of caspase-3 activity was even greater in cells exposed to DHA in combination with T-DM1, suggesting that cell death occurred in mitosis (Figure 6c). On the contrary, a different pattern was found in HCC1954 cells. Indeed, no accumulation of cyclin B1 was found in both T-DM1 and DHA and T-DM1 treated cells. However, the percentage of sub-G1 phase were significantly increased. To confirm the induction of apoptosis, we evaluated the active caspase 3 and the proteolytic cleavage of PARP. Figure 6c shows that T-DM1 treatment induced a significant increase of cell death as evaluated by the amount of cleaved PARP, which was further increased upon T-DM1 and DHA treatment. As DNA damage triggers PARP activation [63], we evaluated the amount of DNA damage levels by studying the increase in the phosphorylation levels of histone H2AX. Intriguingly, a greater DNA damage was found in HCC1954 cells subjected to two-drug combination than those exposed to any single agent, as indicated by the increased levels of γH2AX (Figure 6c). 

Altogether, these data show that DHA in combination with T-DM1 induces severe mitotic defects and death in aggressive HER2+ BC cell lines.

## 4. Discussion 

Microtubule-targeting drugs are extensively used in breast cancer therapy [64]. However, neurotoxicity and the development of resistance represent a clinical problem [65]. 

Spindle-targeting drugs, such as inhibitors of several kinases involved in the formation and function of the mitotic spindle, are in development to improve anti-mitotic chemotherapy [66]. PLK1 plays a crucial role in cell proliferation through its effects on chromosome segregation, spindle assembly, maturation of the centrosome, and cytokinesis during mitosis [27]. PLK1 is highly expressed in several human cancers with poor clinical outcome [67,68,69]. However, if PLK1 overexpression contributes to tumour formation, by inducing mitotic alteration and chromosomal instability, is still a matter of debate [70]. Beyond these conflicting results, PLK1 is currently studied as worthwhile therapeutic target [71]. Notably, the resistance to taxane therapy [71] or to T-DM1 treatment have been linked to PLK1 overexpression in HER2+ BC [72]. However, preclinical success with PLK1 inhibitors, has not translated well into clinical success. Dose-limiting toxicities of PLK1 inhibitors and specificity are critical issues [71].

In accordance with previously reported data, demonstrating TCTP’s role as a key mitotic target of Plk1 in regulating anaphase progression [30] we recently, showed that TCTP is a direct substrate of PLK1 in mammary carcinoma cell lines. We also showed that DHA, by targeting phospho-TCTP, induces apoptosis and enhances the efficacy of chemotherapy and trastuzumab in HER2 overexpressing tumour cells [10]. 

The role of phospho-TCTP in cancer cells has not been thoroughly investigated, and only few studies in the literature show that the phosphorylation of TCTP is required for metaphase-anaphase transition [30,33,73]. In addition, high levels of phospho-TCTP are associated with adverse prognostic factors in breast cancer patients and in neuroblastoma patients, as previously reported by our group [10] and by Ramani et al [74], respectively. Moreover, we also showed that an increase of the nuclear phospho-TCTP level is associated with a poor clinical response to trastuzumab therapy in HER2-positive breast cancer [10]. 

In this study, we show that the reduction of phospho-TCTP levels induced by DHA produces a phenotype resembling almost that observed following PLK1 inhibition. As cellular models for our studies, we chose the HCC1954 and the HCC1569 cell lines, which resemble HER2 overexpressing tumours with *PI3KCA* mutation and loss of PTEN, respectively. In addition, both cells lines were resistant to trastuzumab therapy. Interestingly, malignant progression of HER2-positive breast cancer is often characterized by aberrant PI3K/AKT activation [1,75,76]. For instance, *PI3KCA* mutations and a high proliferation rate are unfavourable prognostic factors in relapsed and de novo metastatic HER2-positive breast cancers treated with trastuzumab [77]. In addition, the loss of at least one copy of the *PTEN* gene is associated with a poor worse outcome in HER2-positive breast cancer, although it is not yet clear whether it is predictive of trastuzumab resistance [78,79].

The levels of phospho-TCTP are critical during mitotic process. Indeed, MCF10A cells overexpressing the non-phosphorylatable form of TCTP showed numerous anomalies of the mitotic spindle. On the contrary, overexpression of the phosphorylatable form of protected MCF10A cells from aberrant mitosis. These data suggest that the reduction of phospho-TCTP levels could be deleterious for achieving proper cell division in growing conditions (Figure 3). In MCF10 cells overexpressing non-phosphorylatable form of TCTP we found high levels of aberrant mitosis. Abnormal mitosis in these cells occurred without any acquisition of DNA damage (Supplementary Figure S2), which in turn may help these cells to bypass surveillance mechanisms. In this context, we can speculate that overexpression of TCTP may protect from oxidative stress that could induce DNA damage in these cells, in accordance with our previously reported data that show TCTP as critical survival factor against oxidative stress [13]. It has been reported that in parasite *Brugia malayi*, TCTP protects DNA from oxidative damage [80]. However, we cannot exclude that DNA damage could occur during mitosis but it is likely to be transient or below a critical threshold necessary to activate p53 to levels that prevent proliferation. These findings have an important clinical value, as aggressive HER2+ BC cells with mutated p53 could be highly sensitive to the effect of DHA.

Remarkably, in line with data from preclinical and clinical studies showing that DHA has good safety profile [39,41,42,51], we also showed that the EC_50_ of DHA was significantly lower in cancer cell lines (<10 µM) than in non-tumourigenic epithelial cells MCF10A (>30 µM) (Table 1). This suggests that DHA has little side effects, thus representing a potentially interesting approach for breast cancer therapy.

DHA is a pro-oxidant agent [53] that increases the cellular levels of ROS and leads to DNA damage, in line with the recent observation that artesunate, whose active metabolite is DHA, induces oxidative DNA lesions and DNA double-strand breaks (DSB) [81,82] (Figure 2). Altogether, these findings have some important implications. First, the reduction of TCTP could contribute to DHA-mediated oxidative damage through the increase of oxidative stress, in line with our previously reported findings [13]. The second is that a critical threshold of DSBs is necessary to prevent mitotic entry [83]. In this context, we can speculate that, in HCC1954 cells, DHA induces DNA damage at an extent below this critical threshold, Therefore, cells can still enter mitosis; thus, contributing to increase genome instability. However, excessive genomic instability has a deleterious effect to the viability of cancer cells, and could be explored for therapeutic purposes. Moreover, the increase of ROS levels in *PTEN*-deficient HCC1569 cells or *PIK3CA* mutant-HCC1954 cells could be their Achilles’ heel, since it has been reported that high levels of active AKT could increase the susceptibility of cancer cells to oxidative stress [84]. PI3K signalling plays a critical role in cell proliferation, mainly through phosphorylation of AKT. In this context, we can speculate that DHA induces metabolic perturbation through the reduction of AKT phosphorylation levels (Figure 2). This effect is further enhanced by T-DM1 (Figure 5). Beyond the induction of severe mitotic perturbations, the inhibition of pAKT by the treatment may contribute to the successful outcomes of DHA and T-DM1 therapy. These data may have clinical relevance since molecular alterations involving the PI3K/AKT pathway are frequently observed in advanced HER2+ BC.

By reducing phospho-TCTP levels, DHA induces both mitotic aberration and the formation of disorganized microtubule structures (Figure 1; Figure 3). Therefore, DHA exacerbated the cytotoxicity of T-DM1 as inhibitor of microtubules. Moreover, analysis of the dose reduction index (DRI) for each drug in their combination indicated that addition of DHA to T-DM1 allowed a dose-reduction for T-DM1 in both cell lines. These data have important clinical implication, as it has been reported that patients treated with T-DM1 have more adverse events than those treated with adjuvant trastuzumab [6] (Table 2). 

The combination treatment was more effective in killing breast cancer cells when compared to the effect induced by any single agent. Notably, the in vivo data show that the growth of tumour cells resumed after having achieved a complete response to T-DM1 treatment. Conversely, DHA and T-DM1 treatment improved the long-term efficacy of T-DM1 and induced a severe and irreversible cytotoxic effect, even after treatment interruption (Figure 4; Figure 6).

Collectively, these results suggest that DHA synergizes with T-DM1 in trastuzumab-resistant cell lines. By reducing phospho-TCTP levels, DHA enhances the effects of T-DM1 by increasing its effectiveness as a poison for microtubules, and in turn, may cause a dramatic inhibition of cell growth in aggressive breast cancer resistant to trastuzumab therapy. This new therapeutic protocol could lead to a clinically significant improvement in the response to T-DM1 therapy.

## 5. Conclusions

In our study, we showed that phospho-TCTP is essential for correct mitosis. Reduction of phospho-TCTP levels by DHA causes mitotic aberration and increases microtubule density in trastuzumab-resistant breast cancer cells. In this way, DHA enhances the effect of T-DM1 as a poison for microtubules, leading tumour cells into an unstable state no longer compatible with viability. T-DM1 is already an FDA-approved agent for advanced breast cancer. DHA is an antimalarial drug, already tested for its toxicity, and has been approved by the European Medicines Agency (EMA) for human use. Due to its low cost and high tolerability profile, it is currently undergoing clinical trials in various types of neoplasia. This study provides the rationale for the design of a new clinical protocol.

## Figures and Tables

**Table 1:** Effects of DHA on cell growth in human mammary cells and in HER2+ BC cell lines. MCF10A-pBabe, MCF10A-AATCTP and MCF10A-WTTCTP cells were treated as described in legend 3b. HCC1569 and HCC1954 cells were treated as described in legend 4a. Half-maximal effective concentration (EC_50_) values derived from concentration-response curves to DHA. Values represent the mean ± SD, *n* = 3. Significant differences between EC_50_ values from breast cancer cell lines and MCF10A cell clones are indicated, *** = *p* < 0.01.

Cell Line	EC_50_ (µM)
MCF10A-pBabe	31.65 ± 8.81
MCF10A-AATCTP	65.95 ± 11.08
MCF10A-WTTCTP	133.00 ± 30.45
HCC1569	8.50 ± 1.70 ***
HCC1954	7.61 ± 1.86 ***

**Table 2:** DHA in combination with T-DM1 causes synergistic inhibition of growth in HER2+ BC cancer cell lines. Cell viability was assessed by ATP assay in cells treated as described in legend 4a. Fractional inhibition = fraction decreased cell viability after treatment, control cells were set to “1”. The parameters m, Dm, and r are the shape of the dose-effect curve, the potency (EC_50_), and the conformity of the data to the mass-action law, respectively. CI values below 0.9 indicate synergistic effect. CI values = 1 indicate additive effect. DRI (DHA) and DRI (T-DM1) are the dose reduction index for DHA and T-DM1, respectively. Value represent the mean ± SD, n = 3.

**HCC1954 cells**						**HCC1569 cells**					
**DHA (µM)**		**Fractional inhibition**	**T-DM1 (µg/mL)**	**Fractional inhibition**		**DHA (µM)**		**Fractional inhibition**	**T-DM1 (µg/mL)**	**Fractional inhibition**	
(D_1_)			(D_2_)			(D_1_)			(D_2_)		
1		0.10 ± 0.08	0.01	0.04 ± 0.03		1.25		0.11 ± 0.06	1	0.03± 0.04	
2.5		0.15 ± 0.09	0.1	0.22 ± 0.07		2.5		0.26 ± 0.07	2.5	0.09 ± 0.07	
5		0.29 ± 0.14	0.25	0.38 ± 0.15		5		0.41 ± 0.06	5	0.13 ± 0.08	
10		0.61 ± 0.16	0.5	0.68 ± 0.13		10		0.54 ± 0.05	10	0.23 ± 0.02	
20		0.85 ± 0.08	1	0.78 ± 0.07		20		0.65 ± 0.03	20	0.21 ± 0.12	
									50	0.57 ± 0.11	
									200	0.59 ± 0.04	
Parameter			Parameter			Parameter			Parameter		
EC_50_ (µM)		7.61 ± 1.86	EC_50_ (µg/mL)	0.33 ± 0.18		EC_50_ (µM)		8.5 ± 1.7	EC_50_ (µg/mL)	101.70 ± 44.8	
m		1.88 ± 0.47	m	0.88 ± 0.26		m		0.90 ± 0.13	m	0.78 ± 0.24	
r		0.98 ± 0.01	r	0.96 ± 0.02		r		0.98 ± 0.02	r	0.97 ± 0.01	
(D1) + (D2) Ratio 10:1						(D_1_) + (D_2_) Ratio 1:1					
		Fractional inhibition	CI	DRI (D_1_)	DRI (D_2_)			Fractional inhibition	CI	DRI (D_1_)	DRI (D_2_)
2.5	0.25	0.74 ± 0.08	0.53 ± 0.11	6.1	3.5	2.5	2.5	0.30 ± 0.10	0.35 ± 0.24	1.47	9.32
5	0.5	0.81 ± 0.06	0.77 ± 0.31	4.1	2.65	5	5	0.51 ± 0.02	0.57 ± 0.18	1.84	15.55
10	1	0.90 ± 0.04	0.99 ± 0.53			10	10	0.59 ± 0.03	0.59 ± 0.37	1.28	12.07
20	2	0.94 ± 0.02	1.28 ± 0.80			20	20	0.70 ± 0.01	0.90 ± 0.37	1.06	11.63
(D_1_) + (D_2_) No constant ratio						(D_1_) + (D_2_) No constant ratio					
		Fractional inhibition	CI	DRI (D_1_)	DRI (D_2_)			Fractional inhibition	CI	DRI (D_1_)	DRI (D_2_)
1.25	0.1	0.21 ± 0.10	2.15 ± 0.35			1.25	2.5	0.26 ± 0.03	0.71 ± 0.03	2.39	7.10
1.25	0.25	0.44 ± 0.14	1.14 ± 0.30			1.25	5	0.28 ± 0.01	0.84 ± 0.11	2.66	4.08
2.5	0.1	0.26 ± 0.01	1.80 ± 0.70			2.5	2.5	0.43 ± 0.09	0.56 ± 0.13	2.64	20.00
2.5	0.25	0.64 ± 0.14	0.48 ± 0.17	4.35	2.16	2.5	5	0.44 ± 0.07	0.58 ± 0.02	2.75	10.61
2.5	0.5	0.74 ± 0.11	0.64 ± 0.14	6.18	1.75	2.5	10	0.45 ± 0.08	0.59 ± 0.13	2.87	5.60
5	0.1	0.40 ± 0.06	1.51 ± 0.45			5	2.5	0.50 ± 0.03	0.63 ± 0.11	1.76	29.45
5	0.25	0.75 ± 0.17	1.60 ± 0.45			5	5	0.50 ± 0.06	0.67 ± 0.13	1.76	14.72
5	0.5	0.79 ± 0.09	0.96 ± 0.31			5	10	0.54 ± 0.07	0.61 ± 0.13	2.08	9.15

## Supplementary Materials

The following are available online at https://www.mdpi.com/2073-4409/9/5/1260/s1, Figure S1: Effect of trastuzumab and pertuzumab on cell growth of HER2+ BC cell lines, Figure S2: DHA induces an increase of ROS levels in MCF10A-pBabe, MCF10A-AATCTP and MCF10A-WTTCTP cells, Figure S3: In vivo efficacy of DHA with T-PM1 in HCC1954 xenografts, Figure S4: Effect of two-drug combination on microtubule density in HCC1569 and HCC1954 cells.
